# Supplementary material for: Smart hybrid microscopy for cell-friendly detection of rare events
Source: Nat Commun. 2026 Jan 7;17:1423. doi: 10.1038/s41467-025-68168-4 (PMC12881448; doi:10.1038/s41467-025-68168-4)
Supplement: Supplementary file 1 — Supplementary Information [file 41467_2025_68168_MOESM1_ESM.pdf]

Supplementary Information

1 Phototoxicity of imaging modalities

To quantify the potential improvement achievable by our smart acquisitions, we performed phototoxicity experiments comparing different imaging modalities. We interleaved the measurements for all modalities on the same sample to reduce bias in the comparisons (Supplementary Figure 1a), and stained the sample with a Hoechst dye and SYTOX orange to quantify cell death. The Hoechst channel allowed for segmentation of the nuclei, while SYTOX orange overlapped with the nuclei regions was used to signal the early stages of cell death. For fluorescence and phase contrast, we took 249 frames with 100 ms exposure at 1 fps for two different fluorescence illumination intensities, and imaged the sample in all channels every 250 seconds for all conditions (Supplementary Figure 1b).

To assess the image quality corresponding to the phototoxicity of a certain condition, we quantified signal-to-noise ratio (SNR) and contrast (Supplementary Figure 1b). Due to the different characteristics of the images in phase contrast and fluorescence, we opted for a manual labeling approach for mitochondria as foreground and adjacent regions as background that did not show distinctive features in phase contrast. Metrics were calculated by comparing the data in these regions for the first 1000 frames and absolute values are reported in the corresponding panels. SNR was calculated as mean foreground intensity over standard deviation of the background and contrast as mean foreground intensity minus mean background intensity.

In phase contrast and dark conditions, we detected less than 10% cell death across experiments. The results were indistinguishable from one another and similar for both SNR and contrast. On the other hand, using typical fluorescent imaging conditions for MitoTracker (35 mW/mm^2), we observed noticeable cell death already at the first measurement timepoint, reaching a half-life time of 8.5 minutes when fitting an exponential decay curve (Supplementary Table 1). This results in a decay rate ratio $\lambda_{fluo}/\lambda_{phase}$ of 53 and an excess mortality in fluorescence imaging $(\lambda_{fluo} - \lambda_{dark})/(\lambda_{phase} - \lambda_{dark})$ of 4800. Lowering the laser intensity to 8 mW/mm^2 reduced the cell death phenotype, still reaching 40% after an hour, with a marked loss in image quality (SNR and contrast).

Taken together, our data suggest that while fluorescence presents a trade-off between image quality and cell death even at low-dose widefield imaging conditions, phase contrast imaging can be performed at high framerates during long acquisitions with minimal cell toxicity and image quality loss.

Method	Decay Rate 1/h	Half-Life Time
Fluorescence (10 %)	4.9	8.5 mins
Phase Contrast	0.091	7.6 h
Dark	0.090	7.7 h

Supplementary Table 1| Decay rates and half-life times for each condition.

2 Inference time and latency assessment

We measured both the inference times for each presented model and the latency until the subsequent smart frame acquisition, by analyzing data from 1000-frame time-lapse experiments. For the mitochondrial fission model, the average prediction time was 192.8 ± 20.0 ms (n = 996), while the average latency between the event detection and the acquisition of the successive smart frame was 1889.9 ± 37.7 ms (n = 99). For the Lipid Droplets–mitochondria interactions, the average prediction time was 168.3 ± 25.3 ms (n = 998), and the average smart-frame latency was 1892.1 ± 43.2 ms (n = 99). Reported uncertainties correspond to sample standard deviations. The latency between detection and smart-frame acquisition arises because smart frames are currently attached to the next scheduled acquisition. In our experiments, the frame-rate is set at 1 Hz, keeping a stable frame rate for easy analysis, but leading to delays on the order of one second. In principle, this interval could be significantly shortened by triggering the smart acquisition immediately after the event detection, rather than waiting for the next frame. Thus, the primary limiting factor in this case would be the model inference time, which could be easily improved, e.g., by using faster hardware.

3 Deep Events data handling

In order to handle the data for this project, we have established a database-backed data ingestion pipeline (Supplementary Figure 2). It handles data from different sources (microscopes/softwares) and samples and organizes both image and metadata. Specific to our application is the integration of time lapses and sparse annotations. The balance between negative and positive instances in the training data is important for the training process. As events are randomly distributed, we crop events from the full-frame time lapses in space and time. Additionally we add

sequences that do not contain events as negatives. Extracted sequences and the corresponding metadata are added to a central database, which can be queried for combinations of data (frame rates, illumination settings, etc.). For training, a prompt into the database produces corresponding training and evaluation datasets. Together with the model, we save the metadata for the training (prompt, model parameters, etc.) and the performance data (scores, prediction histograms, etc.). To optimize subsequent trainings, we have implemented a manual curation workflow that enables addition of training data to the database when one of the models is used. False positives indicate valuable training data for the model to become more precise for example. As data is added to the database continuously, we have implemented an automatic re-training procedure that trains models on a weekly basis.

4 Software structure

Commercial microscope software is implemented with the traditional linear approach to multi-dimensional acquisitions (MDA), where a predefined time series of instructions is sent to the microscope for acquisition. Adaptive acquisitions, as we have implemented them here, are not supported by this approach. We have therefore worked together with the pymmcore-plus team to implement adaptive acquisitions in the pymmcore-plus framework[1,2]. The surveillance acquisition is sequenced by a slightly adapted MDARunner that uses the MDAEngine to relay MDAEvents to the microscope via the pymmcore (Python) bridge to the Micro-Manager core (C++). The microscope (camera) returns the images and an event is published in the events backend in pymmcore-plus. Our on-the-fly analysis pipeline subscribes to this event to be notified for the arrival of new data (Supplementary Figure 4).

A component called analyser handles the data ingestion, stacking time frames for the model and filtering data from other channels. It implements preprocessing of the frames and runs inference of the model once enough frames have arrived. The resulting event score map is processed, and a final event score for the current time point is sent to an interpreter. The interpreter uses this data to send the decision to activate or deactivate the additional channel(s) to the actuator. It implements memory, allowing advanced settings like a minimal number of frames to be taken in the smart acquisition settings. The actuator stores the additional acquisition settings and changes the active acquisition sequences in the smartRunner. With this, the loop is closed and the runner is informed which events to send to the MDAEngine at any given time point.

Relevant code:

- Components (analysers, interpreters, actuators)
- Actuator used for the modality switch
- Script to run the software
- Training

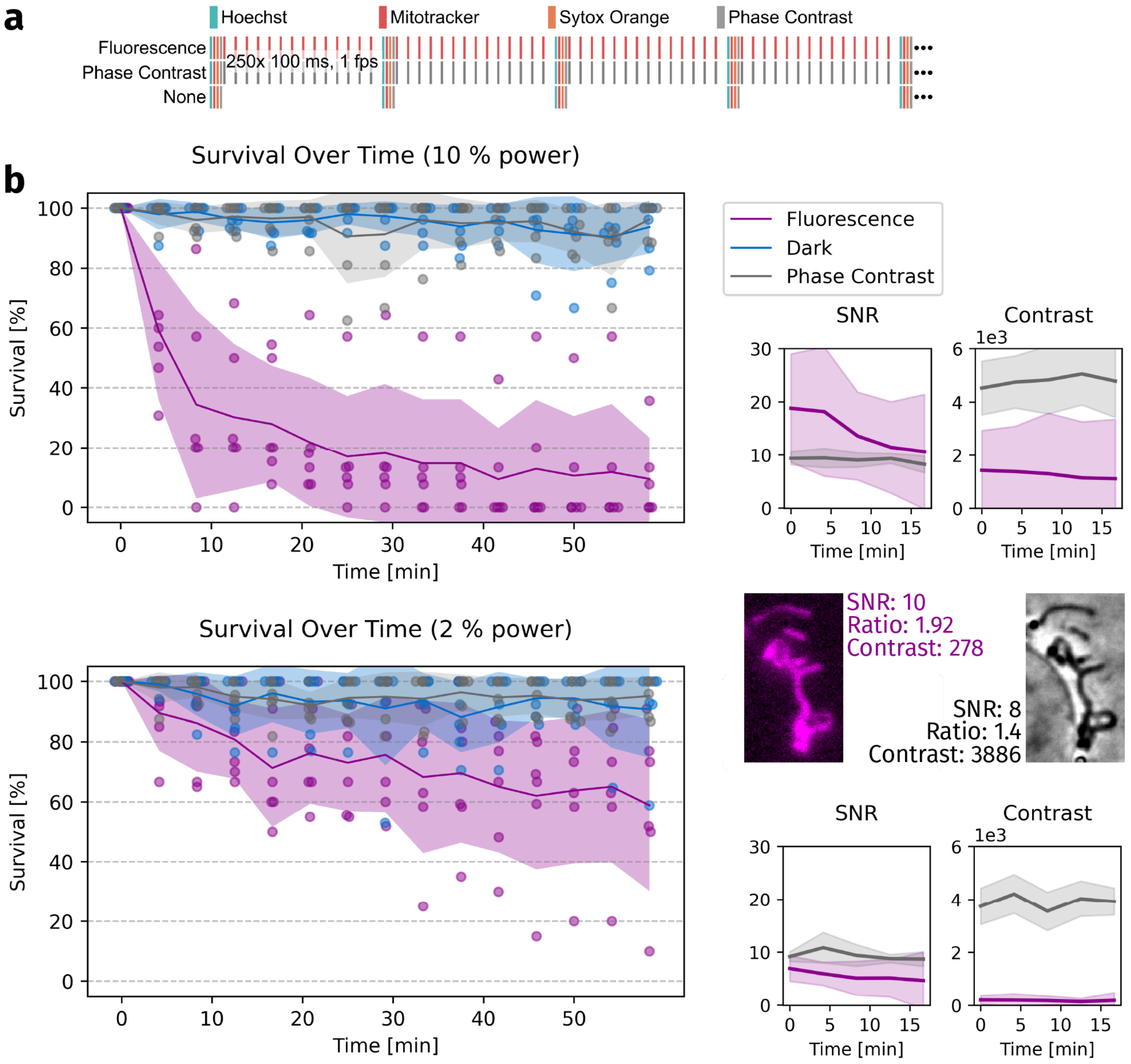

Supplementary Figure 1| Label-free acquisition causes greatly reduced levels of phototoxicity compared to fluorescence imaging. a, Graphic depiction of imaging setup, vertical lines represent acquired images. b, Survival plots for SYTOX toxicity experiment; each point represents an independent FOV (left), and image quality measures in each experiment, including inset examples with measured values (right). Shaded lines represent mean ± sd. n=18 measurements from 3 independent samples. Source data are provided as a Source Data file.

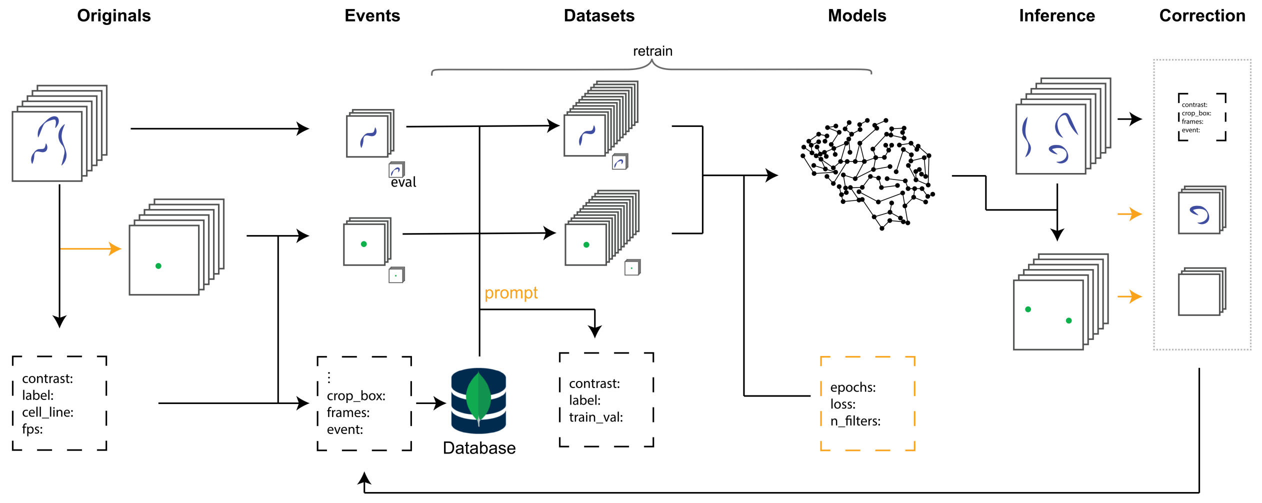

Supplementary Figure 2| Overview of the data flow for the deep-events package. Raw microscopy data flows through event extraction, database storage with metadata integration, and dataset generation for model training. A central database enables balanced sampling of positive/negative instances, with automatic re-training procedures ensuring model improvement over time.

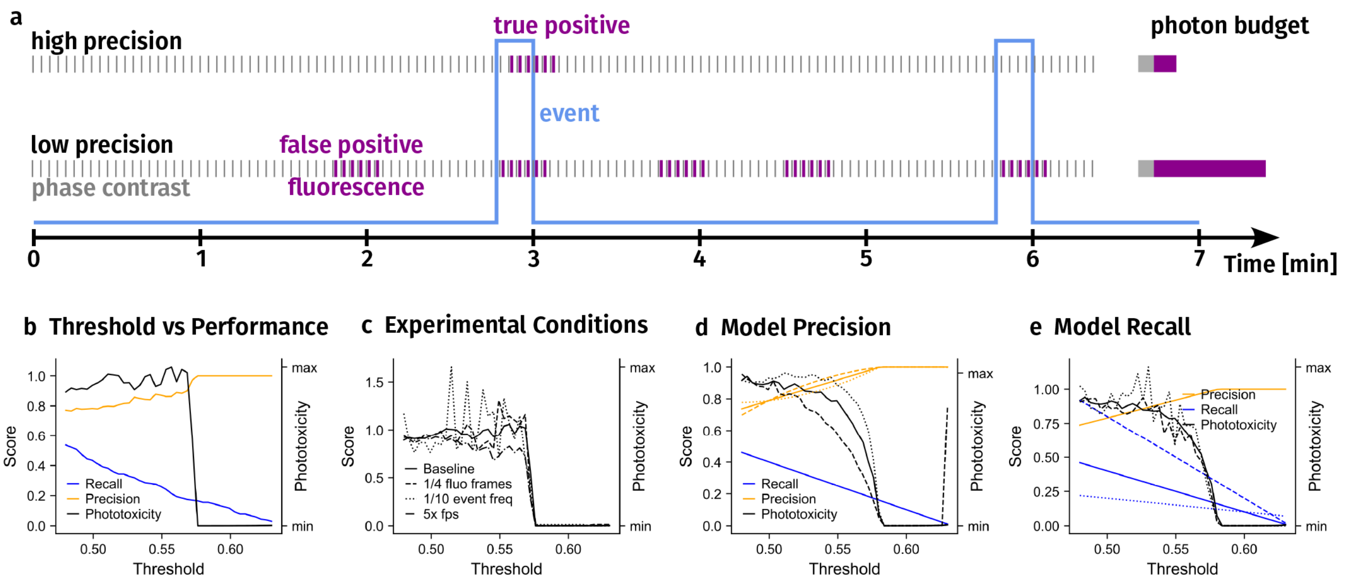

Supplementary Figure 3| Hybrid-EDA performance with different model and experimental conditions. a, Schema of simulations of hybrid-EDA experiments. Precision and recall were extracted from model validation data (b, c) or simulated (d, e). With a defined event signature (blue), at each phase-contrast frame there is a certain probability for the framework to activate fluorescence frames. This probability is determined by the state of the sample. If no event is ongoing, the precision of the model determines if a false positive triggers fluorescence imaging; low precision leads to a high number of false positives. If an event is ongoing, the recall probability determines if the event is captured by activating fluorescence imaging; low recall results in more missed events. In order to assess imaging impact, acquired frames were counted in both modalities, weighted by their relative excess mortality (4800 fluorescence/phase-contrast) determined in Suppl. Note 1, and summed to a final phototoxicity score. This value was then normalized by the number of captured events to give a performance score for the hybrid-EDA acquisition b, Recall and precision for a real model and the photon budget spent to acquire 1000 events, depending on the threshold chosen for activation of fluorescence imaging. c, hybrid-EDA performance at different thresholds for a variety of experimental conditions. Even extensive changes in conditions show only slight differences in threshold behavior. Baseline settings were simulated to mirror the experimental conditions used in the experiments (1Hz, 20 fluorescence frames, 1 event every 3 minutes, predictable for 5 seconds) d, Hybrid-EDA performance is sensitive to the precision of the model and choice of threshold. This is by design. Hybrid-EDA is optimized for capturing rare events, a lower threshold results in lower precision, which leads to many false positives because of the class imbalance between the rare events and the ‘normal’ state of the sample. These false positives all contribute to the phototoxicity of the acquisition, lowering the effectiveness of hybrid-EDA. Depending on the specific precision behavior of the model at different thresholds, however, a trade-off between hybrid-EDA performance and recall can be made. e, Differences in recall do not have a big influence on hybrid-EDA performance. The code to generate these plots can be found in the supplementary data, as well as the recall and precision curves for all models used in the hybrid-EDA experiments. Source data are provided as a Source Data file.

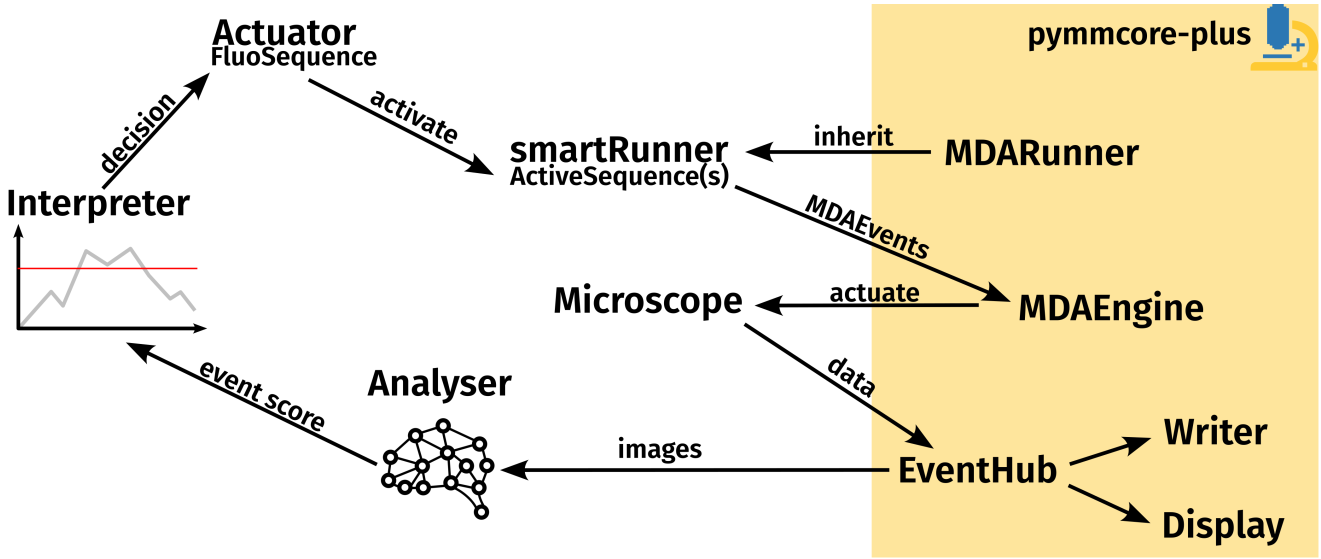

Supplementary Figure 4| Graphical representation of the software structure. The software components interact with pymmcore-plus via efficient event-based communication. Event scores are calculated from images by the analyser. The interpreter contains long-term temporal context and uses this information to decide on hybrid-EDA activation. Switching between modalities is implemented by the actuator and smartRunner. Pymmcore-plus is used for the basic functionality of microscope control, visualization, and data output.

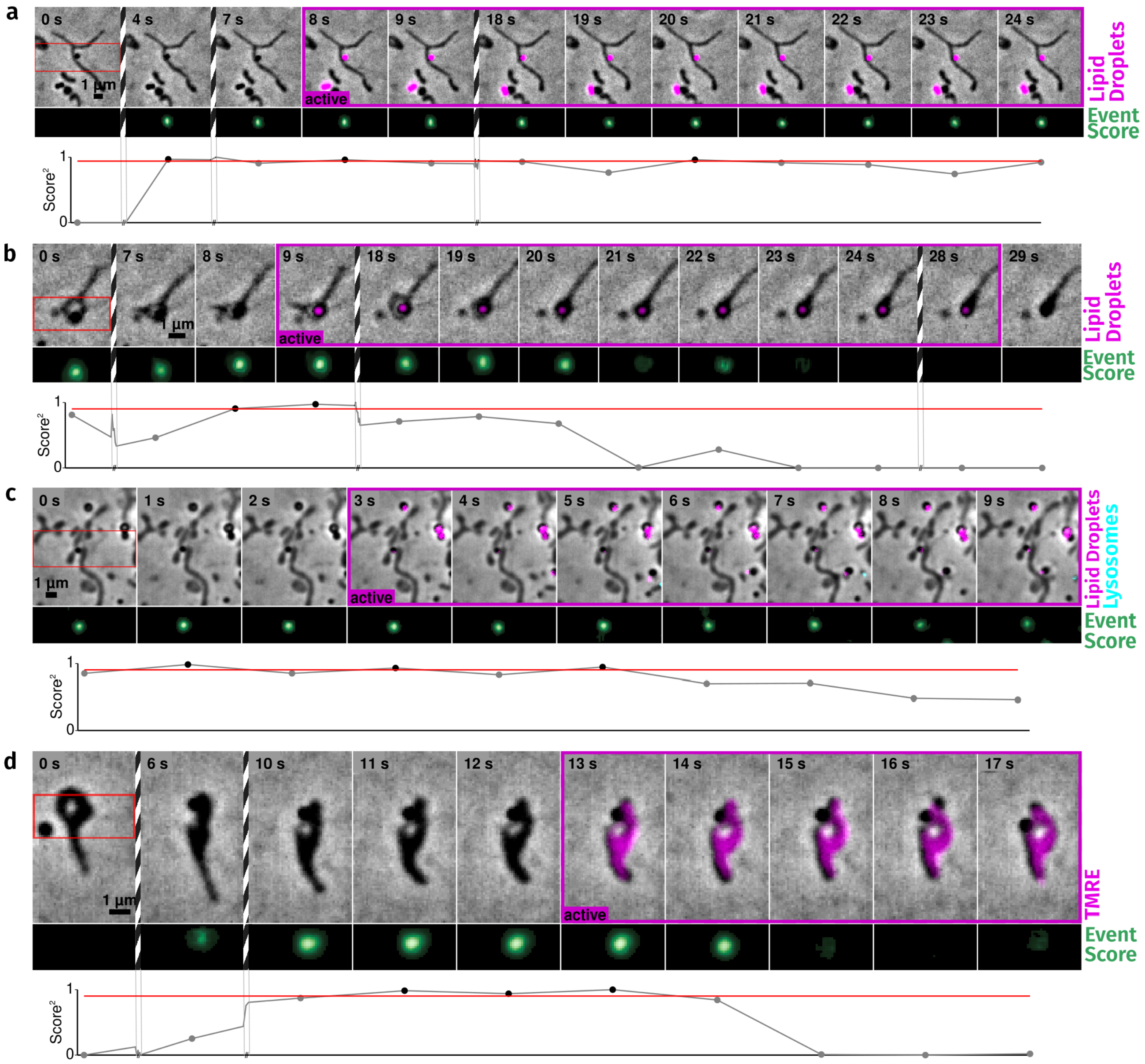

Supplementary Figure 5| Additional examples of organelle contact events acquired by hybrid-EDA microscopy. Phase-contrast imaging overlaid with fluorescence, as labeled on the right. Below each image time series is the score obtained from the event detection model as an image (crop marked as a red square at time 0) and an integrated plot. The red line indicates the trigger threshold; vertical striped lines indicate cropped timepoints. The corresponding time-lapses are shown in Supplementary Movies 2, 3, 5, and 6.

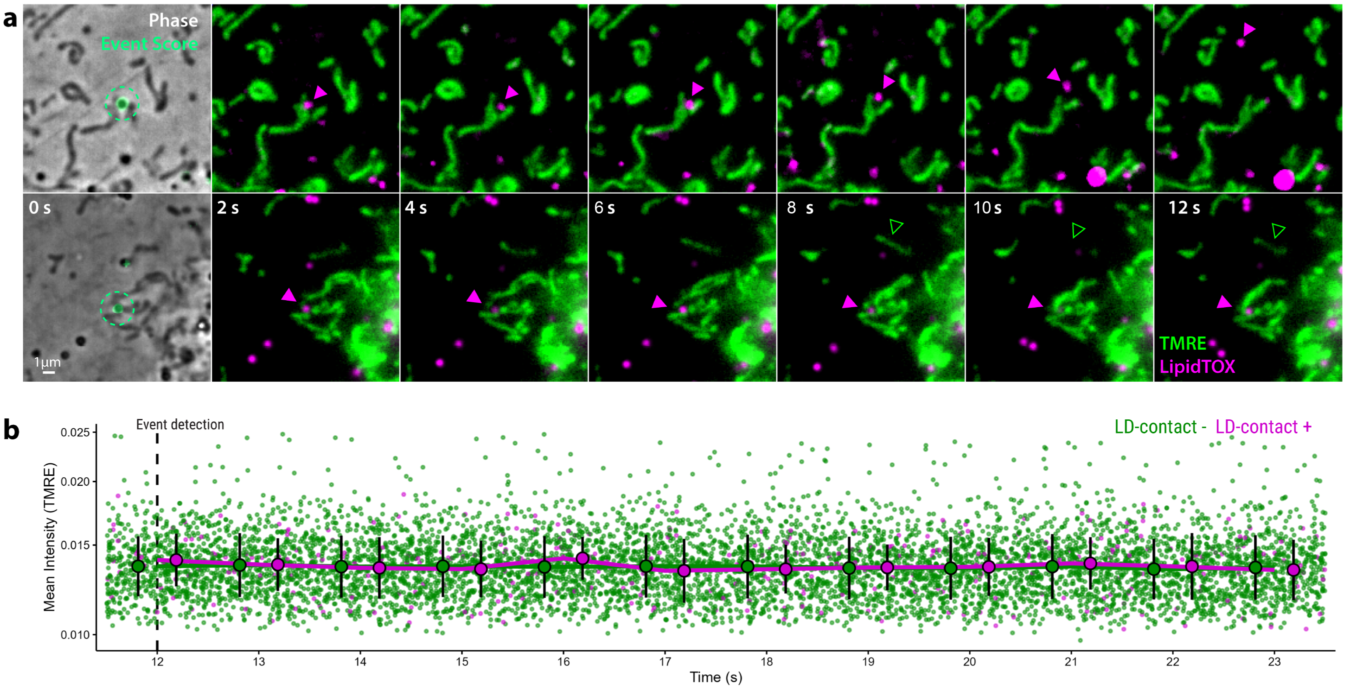

Supplementary Figure 6| Mitochondrial membrane potential quantification during Lipid Droplet–mitochondria contacts. a, Representative time-lapses of detected events. Time 0 shows the phase-contrast and model-detected score overlaid, while the rest of the time-lapse displays the triggered double fluorescence imaging of lipid droplets (LD, LipidTOX, magenta) and mitochondrial membrane potential (TMRE, green). Magenta arrowheads follow the triggering lipid droplet, while the empty green arrowhead indicates a mitochondrion spontaneously "flickering" membrane potential. Dashed circle indicates the area used for LD–mitochondrial proximity classification at time = 0. b, Quantification of membrane potential by mean TMRE intensity (arbitrary units) for individually segmented mitochondria, classified by whether they were proximal to the detected contact or not (magenta and green, respectively). A total of n = 459 mitochondria were quantified across 16 movies (∼13 frames each), of which 30 were LD^+ and 429 were LD^-. Source data are provided as a Source Data file.

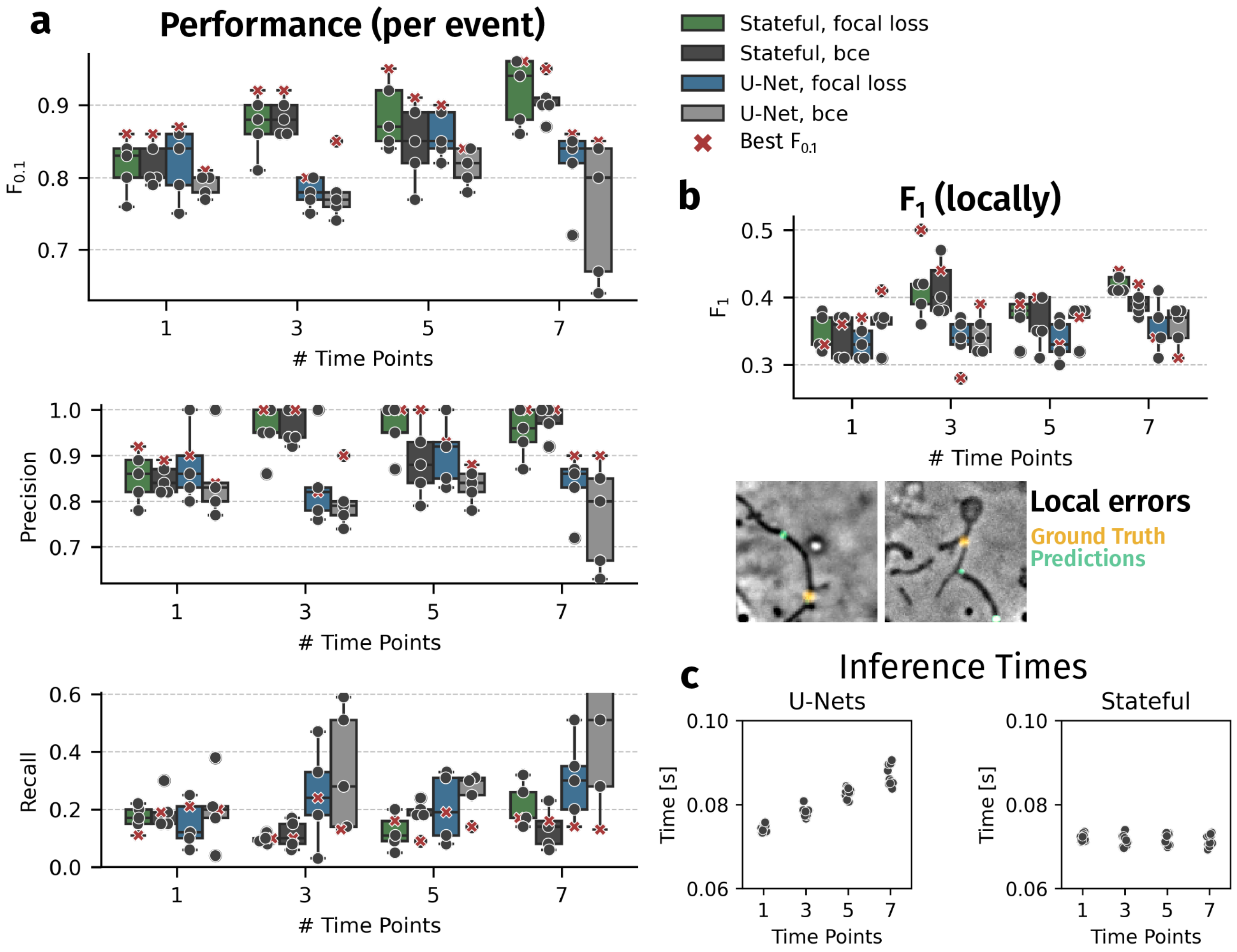

Supplementary Figure 7| Neural network training for the detection of division pre-states in mitochondria was performed with the different loss functions and architectures presented in this work. a, Main performance scores calculated for all combinations of architectures and loss functions on an event basis (detection true/false per 5 frames). b, Local F_1 scores show improvements with additional time points for all loss functions, in particular for the stateful architectures. Relatively low scores can be explained by detection of constrictions on a mitochondrion that is prone to divide, but divides at a position whose event score is not the global maximum. These local false positives are not detrimental to hybrid-EDA, since a trigger on the correct mitochondrion will still acquire the event of interest. c, The prediction time of U-Nets increases slightly with more time points due to more data being handled at the same time. As the state in stateful U-Nets can be saved in the layer itself, every individual inference only needs one frame at a time, leading to a constant inference time for on-the-fly processing of a continuous image stream. Each point indicates the performance of a separate model, with an X indicating the best-performing one. Box plots mark the first quartile, median, and third quartile with the whiskers spanning the 5th and 95th percentile. Source data are provided as a Source Data file.

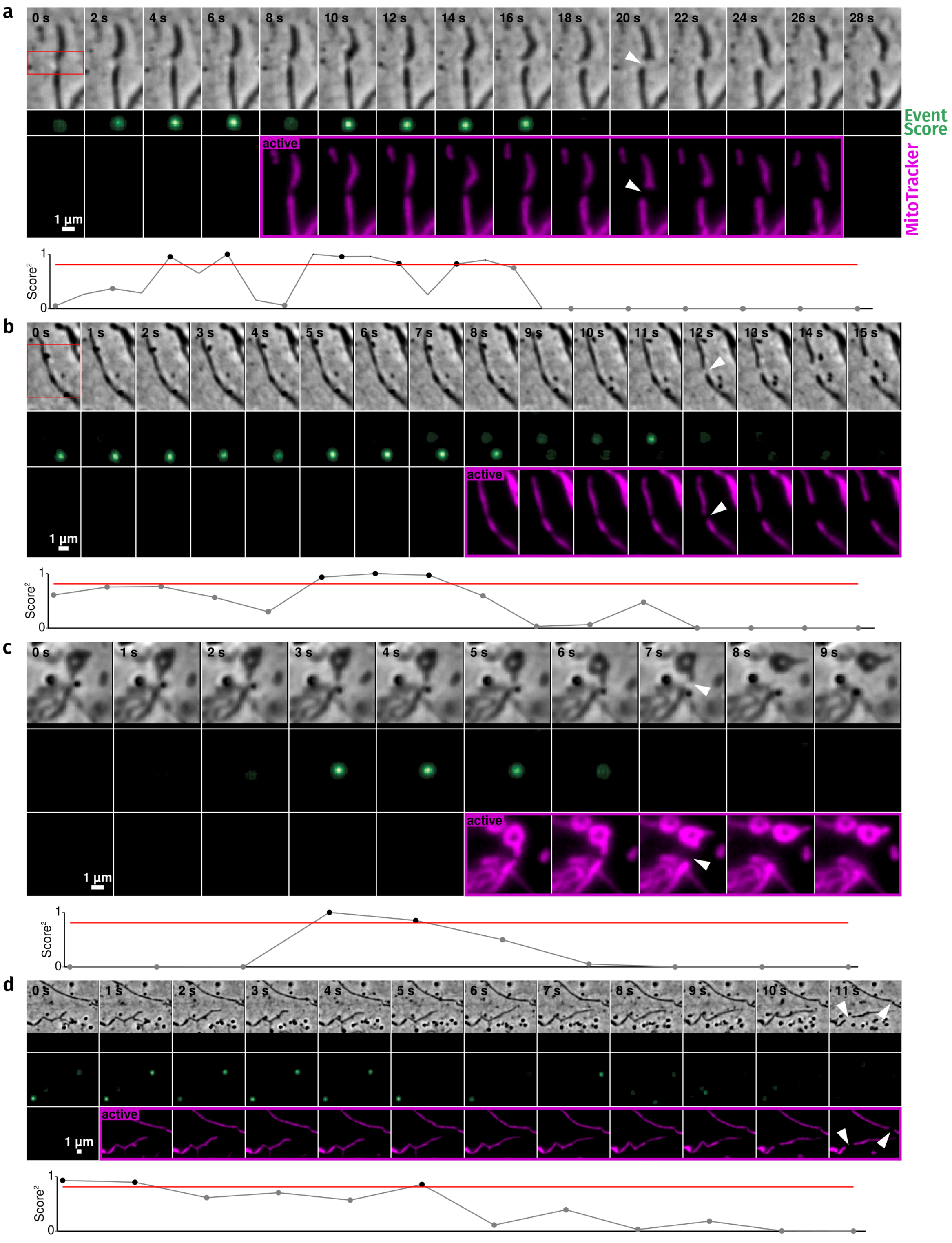

Supplementary Figure 8| Mitochondrial division events captured using hybrid-eda. A variety of mitochondrial morphologies triggered the adaptive fluorescence acquisitions in different cellular environments. Phase-contrast imaging (top), MitoTracker fluorescence (bottom), and score obtained from the model on-the-fly as an image (crop marked as a red square at time 0). The event score is plotted below each series with a red line marking the threshold used. Capturing the fluorescence channel during the divisions allows for the use of established analysis tools for segmentation and quantification compared to the very limited tool set available for phase-contrast data. This allows for analysis of mitochondrial length, diameter, and network structure during division events. The corresponding time-lapses are shown in Supplementary Movies 7–10.

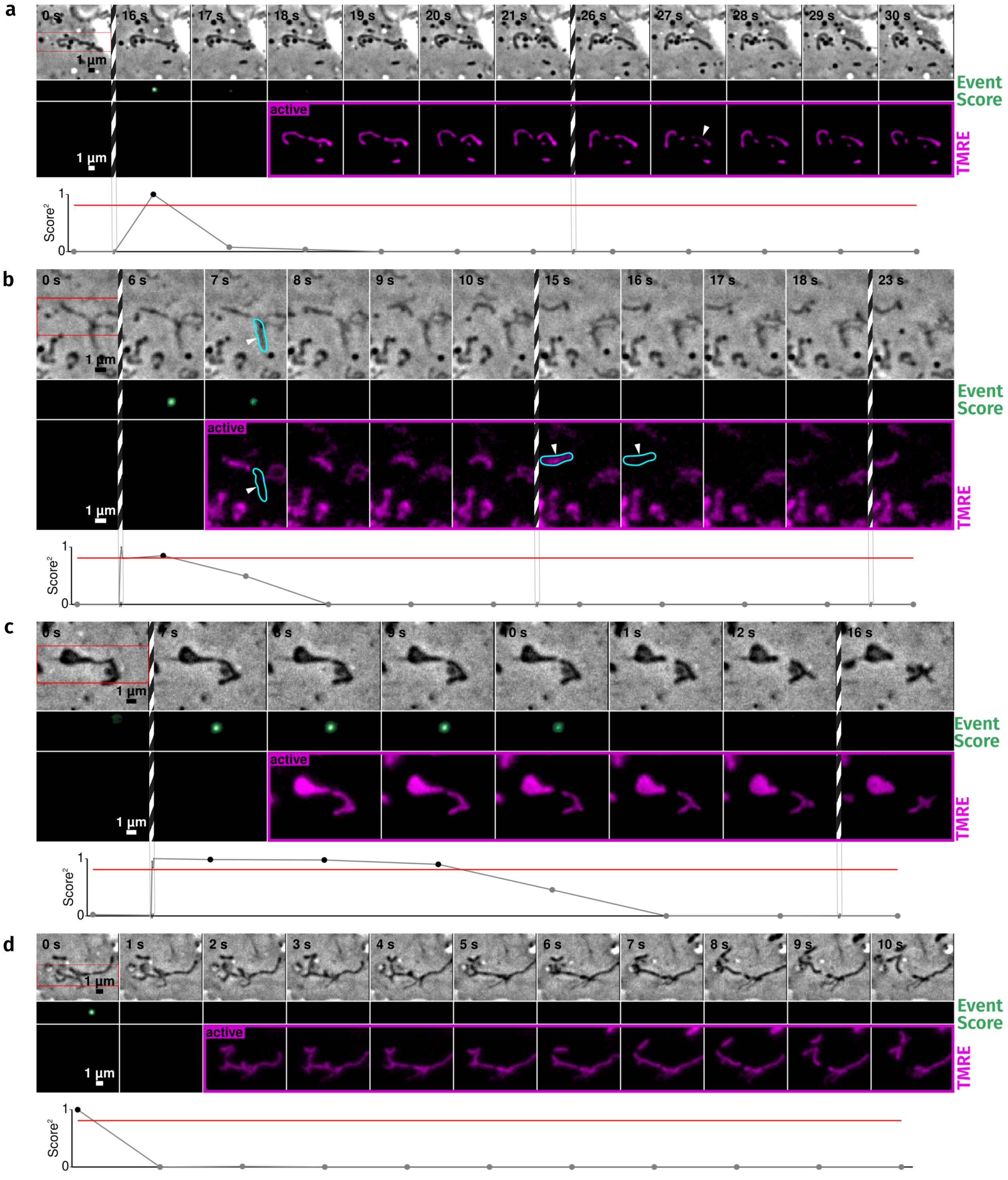

Supplementary Figure 9| Mitochondrial membrane potential during division events captured using hybrid-eda. A variety of mitochondrial morphologies triggered the adaptive fluorescence acquisitions in different cellular environments. Phase contrast imaging (top), TMRE fluorescence (bottom), and score obtained from the model on-the-fly as an image (crop marked as a red square at time 0). The event score is plotted below each series with a red line marking the threshold used. In panel b, the cyan contour highlights the outline of the relevant part of the mitochondrion before and after membrane potential loss. The corresponding time lapses are shown in Supplementary Movies 13–16.

References

1. Lambert, T., gasparoli, f., Hunt-Isaak, I., Stepp, W. L. & hinderling. *Pymmcore-plus/Useq-Schema: V0.7.0* Zenodo. Feb. 2025.
2. Lambert, T. *et al. Pymmcore-plus/Pymmcore-plus: V0.13.2* Zenodo. Feb. 2025.
