# Supplementary material for: Smart hybrid microscopy for cell-friendly detection of rare events
Source: Nat Commun. 2026 Jan 7;17:1423. doi: 10.1038/s41467-025-68168-4 (PMC12881448; doi:10.1038/s41467-025-68168-4)
Supplement: Supplementary file 3 — Description of Additional Supplementary Files [file 41467_2025_68168_MOESM3_ESM.pdf]

Supplementary Movies

Supplementary Movies 1, 2, 3: examples of mitochondria-lipid droplets contact events. Representative smart hybrid time-lapses (65, 26 and 31 frames, 1Hz) of organelle contact events in COS-7 cells stained with the lipid droplet marker mEmerald-PLIN5. Left: phase contrast (gray), overlaid with the above-threshold event score output from the network (orange). Right: the triggered fluorescence acquisition of the lipid droplets (magenta). Representative frames from Supplementary Movies 1, 2 and 3 are shown in Fig. 2e, Supplementary Figure 5a and b, respectively. Scale Bar: 10 µm.

Supplementary Movies 4, 5: examples of mitochondria-lipid droplets and mitochondria-lysosomes contact events. Representative smart hybrid time-lapses (37 and 33 frames, 1Hz) of organelle contact events in COS-7 cells stained with the lipid droplet marker LipidTOX and the lysosome marker LysoTracker Red. Left: phase contrast (gray), overlaid with the above-threshold event score output from the network (orange). Right: the triggered fluorescence acquisition of the lipid droplets (magenta) and lysosomes (cyan). Representative frames from Supplementary Movies 4 and 5 are shown in Fig. 2f and Supplementary Figure 5c, respectively. Scale Bar: 10 µm.

Supplementary Movie 6: example of mitochondrial membrane potential during a mitochondrion-lipid droplet contact event. Representative smart hybrid time-lapse (31 frames, 1Hz) of mitochondrial membrane potential in COS-7 cells stained with TMRE. Left: phase contrast (gray), overlaid with the above-threshold event score output from the network (orange). Right: fluorescence acquisition of the membrane potential (magenta) as revealed by TMRE, triggered by the detected mitochondrion-lipid droplet contact event. Representative frames from Supplementary Movie 6 are shown in Supplementary Figure 5d. Scale Bar: 10 µm.

Supplementary Movies 7, 8, 9, 10: examples of mitochondrial divisions detected in phase contrast time-lapses by the U-Net. Representative smart hybrid time-lapses (35, 55, 25, 37 frames, 1Hz) of mitochondrial divisions detected by the U-Net in COS-7 cells stained with MitoTracker. Left: phase contrast (gray), overlaid with the above-threshold event score output from the network (orange). Right: the triggered fluorescence acquisition of the MitoTracker signal (magenta). Representative frames from Supplementary Movie 7, 8, 9 and 10 are shown in Supplementary Figure 8a, b, c and d, respectively. Scale Bar: 10 µm.

Supplementary Movie 11: example of a mitochondrial division and related DRP1 dynamics. Smart hybrid time-lapse (54 frames, 1Hz) of a mitochondrial division in a COS-7 cell stained with DRP1-mEmerald. Left: phase contrast (gray), overlaid with the above-threshold event score output from the network (orange). Right: the triggered DRP1 fluorescence acquisition (magenta). Representative frames from Supplementary Movie 11 are shown in Fig. 3f. Scale Bar: 10 µm.

Supplementary Movies 12, 13, 14, 15, 16: examples of mitochondrial membrane potential during division events. Representative smart hybrid time-lapse (42, 52, 41, 56, 29 frames, 1Hz) of mitochondrial membrane potential in COS-7 cells stained with TMRE. Left: phase contrast (gray), overlaid with the above-threshold event score output from the network (orange). Right: fluorescence acquisition of the membrane potential (magenta) as revealed by TMRE, triggered by the detected mitochondrial divisions. Representative frames from Supplementary Movies 12,13, 14, 15, 16 are shown in Fig. 3h, Supplementary Figure 9a, b, c and d, respectively. Scale Bar: 10 µm.
